# Combined Effect of Healthy Lifestyle Factors and Risks of Colorectal Adenoma, Colorectal Cancer, and Colorectal Cancer Mortality: Systematic Review and Meta-Analysis

**DOI:** 10.3389/fonc.2022.827019

**Published:** 2022-07-22

**Authors:** Jiazhou Yu, Qi Feng, Jean H. Kim, Yimin Zhu

**Affiliations:** ^1^ Jockey Club School of Public Health and Primary Care, The Chinese University of Hong Kong, Hong Kong SAR, China; ^2^ Nuffield Department of Population Health, University of Oxford, Oxford, United Kingdom; ^3^ Department of Epidemiology & Biostatistics, and Department of Respiratory Diseases of Sir Run Run Shaw Hospital, Zhejiang University School of Medicine, Hangzhou, China; ^4^ Cancer Center, Zhejiang University, Hangzhou, China

**Keywords:** colorectal, lifestyle, index, incident, dose-response, prevention

## Abstract

**Background:**

In addition to adiposity, lifestyle factors such as poor diet, low physical activity, alcohol intake and smoking are noted to be associated with the development of colorectal cancer (CRC). This study aims to investigate the association and dose-response relationship between adherence to a healthy lifestyle and CRC risk.

**Methods:**

A systematic literature search was conducted in MEDLINE and EMBASE for studies examining multiple lifestyle factors with risk of CRC, incident colorectal adenoma (CRA), and CRC-specific mortality through June 2021 without restrictions on language or study design. Meta-analysis was performed to pool hazard ratios using random-effects model. Subgroup analyses were performed based upon study and sample characteristics. Random-effects dose-response analysis was also conducted for CRC risk to assess the effect of each additional healthy lifestyle factor.

**Results:**

A total of 28 studies (18 cohort studies, eight case-control studies, and two cross-sectional study) were included. When comparing subjects with the healthiest lifestyle to those with the least healthy lifestyle, the pooled HR was statistically significant for CRC (0.52, 95% CI 0.44-0.63), colon cancer (0.54, 95% CI 0.44-0.67), rectal cancer (0.51, 95% CI 0.37-0.70), CRA (0.39, 95% CI 0.29-0.53), and CRC-specific mortality (0.65, 95% CI 0.52-0.81). The pooled HR for CRC was 0.91 (95% CI: 0.88-0.94) for each increase in the number of healthy lifestyles. The inverse association between healthy lifestyle and CRC risk was consistently observed in all subgroups (HR ranging from 0.26 to 0.86).

**Conclusions:**

Adoption of a higher number of healthy lifestyles is associated with lower risk of CRC, CRA, and CRC-specific mortality. Promoting healthy lifestyle could reduce the burden of CRC.

**Systematic Review Registration:**

https://www.crd.york.ac.uk/PROSPERO/display_record.php?RecordID=231398, identifier CRD42021231398.

## Introduction

Globally, colorectal cancer (CRC) ranks the third most commonly diagnosed malignancy and the second leading cause for cancer mortality (1). In 2020, CRC accounted for approximately 1.9 million new cases and more than 935,000 deaths worldwide (1). Its disease burden is projected to continue increasing globally, particularly in regions undergoing rapid industrialization (2). The increased risk of CRC and colorectal adenoma (CRA), one of its primary precancerous lesions (3), is closely linked to a variety of modifiable lifestyle risk factors, including excess adiposity (4–6), physical inactivity (7, 8), high intake of red meat and/or processed meat (9, 10), alcohol consumption (11, 12), and smoking (13); higher intake of dietary fiber, vegetables, and fruits are noted to be protective against CRC and CRA (14–17).

While the associations between CRC and single lifestyle factors have been extensively investigated in previous studies (18–21), far fewer studies have examined the effect of the adherence to a healthy lifestyle, defined as a combination of various modifiable factors. A latest meta-analysis that included 17 studies showed an overall inverse association between combined healthy lifestyle factors and CRC risk (22). However, it remains unclear whether the association differs by study settings or population characteristics and whether the association presents a dose-response relationship. In addition to CRC incidence, healthy lifestyle is also suggested to be related to CRC mortality in both CRC patients and general population (23–25). However, to the best of our knowledge, no systematic review and meta-analysis are available so far on the combined healthy lifestyle in relation to CRC-specific mortality.

Hence, this systematic review aims to investigate the association between adherence to a healthy lifestyle and the risk of CRC, CRA, and CRC-specific mortality, and to examine whether the association is dose-dependent and any potential effect modification by population characteristics.

## Methods

This systematic review was registered on PROSPERO (CRD42021231398) and reported according to the Preferred Reporting Items for Systematic Reviews and Meta-Analysis (PRISMA) (26).

### Data Sources and Search

We searched MEDLINE and EMBASE for relevant studies from their inceptions through June 2021. The search strategy combined three groupings of keywords with their derivatives and synonyms related to the following concepts: 1) combined or integrated effect; 2) lifestyle factors or health behaviors; and 3) colorectal cancer and adenoma. The search terms of these three concepts were combined using the Boolean operator “AND”. More details on search strategy is described in **Supplementary Tables 1** and **2**. The reference lists of eligible studies and relevant reviews were manually searched to identify additional publications. The search strategy did not impose any restriction on language, publication period, or publication status.

### Eligible Criteria and Study Selection

We included epidemiological studies that investigated the association between combined lifestyle factors and colorectal outcomes. The exposure was combination of lifestyle factors, including but not limited to diet, smoking, alcohol consumption, physical activity, overweight/obesity, sleep duration, and others. The primary outcomes were risk of CRC, colon cancer, and rectal cancer. The secondary outcomes included risk of incident CRA, advanced colorectal neoplasia, and CRC-specific mortality. We included cross-sectional studies, case-control studies, and cohort studies. For CRC-specific mortality, we included studies of healthy population or CRC patients, while for the other outcome, the study population should be free of the outcomes at baseline if the study design was prospective cohort.

We excluded studies if they were (1) reviews, protocols, conference abstracts or not peer-reviewed publications, (2) focusing on a single lifestyle factor or a combination of less than three factors, (3) development or validation of prediction models, or (4) not reporting relevant data. For duplicate reports from the same cohort, we only included the report that had examined the largest number of lifestyle factors.

We used a two-step study selection procedure. The title and abstract of all electronically and manually identified records were screened first to identify potentially eligible studies. Second, full texts of the potentially eligible studies were examined for final eligibility. Two authors independently performed the selection process. All disagreements were resolved by discussion with a third reviewer until consensus was reached.

### Data Extraction and Quality Assessment

Data were extracted by using an *a priori* designed form which collected the following information: (1) basic characteristics of study and subjects (e.g. first author, publication year, country, study period, sample size), (2) basic characteristics of participants (e.g. age, gender, ethnicity); (3) methodological characteristics, including study design, exposure definitions, outcome attainment, and follow-up period (for cohort studies) (4) effect estimates for the associations of interest, and (5) other information for quality assessment.

The methodological quality of cohort studies, case-control studies, and cross-sectional studies were assessed by the Newcastle-Ottawa Scale, which covers three domains: selection of participants, comparability of study groups, the ascertainment of exposure (for case-control studies) or outcomes (for cohort studies and cross-sectional studies) (27, 28). A star system, with a maximum of nine stars for cohort studies and case-control studies and ten stars for cross-sectional studies, was used to present the result of quality assessment, with more stars representing higher quality and lower risk of bias. We consider cohort studies and case-control studies high quality if they received more than seven stars, moderate quality if they received five or six stars, otherwise low quality. Cross-sectional studies were considered of high quality if they received more than eight stars, moderate quality if they received six or seven stars, otherwise low quality. Data extraction and quality assessment were performed independently by two authors. Any discrepancy was resolved by discussion until consensus was reached.

### Data Synthesis and Analysis

There has been no universal consensus on the quantification of combined lifestyle factors. Most studies constructed a simple unweighted lifestyle score, where one point was given to each of the present healthy lifestyles, although the exact lifestyle factors may vary across studies; for example, in Carr 2018 (29), Hang 2015 (30), and Kirkegaard 2010 (31). Some studies used weighted lifestyle score, in which the factors were weighted differently; for example, in Harnack 2002 (32) and Romaguera 2015 (33). However, some studies constructed risky lifestyle score that assign points to presence of unhealthy lifestyle habits; for example, in Cho 2019 (34) and Erdrich 2015 (35). In order to keep the directionality consistent with studies examining healthy lifestyle factors, we calculated a new score by deducting the original risky lifestyle score from the total number of the lifestyle factors for the studies that focus on unhealthy lifestyle habits (34, 35). The healthy lifestyle score was either used as a continuous variable (measuring the effect of per 1-unit increase in score) or transformed into a categorical variable (measuring the effect of adherence to healthiest lifestyle relative to the least healthy lifestyle) in original studies. In the originals studies, the five most commonly examined lifestyle factors were: diet, smoking status, alcohol consumption, physical activity level, and body measure. Most studies examined all five factors while others included some of them (see **Supplementary Table 3**).

Effect measures comparing the group with the healthiest lifestyle to the group with the least healthy lifestyle was pooled to present the associations of interest. Hazard Ratios (HR) with 95% confidence intervals (CIs) was the most commonly used as the measure of effect in original studies and was therefore used in this meta-analysis. Odds ratios, where applicable, were transformed into RR using the following formula: RR=OR/[(1-P_0_)+(P_0_*OR)], where P_0_ is the risk of an event in the non-exposed group (36). The transformed RRs and those extracted from some original studies were converted into HR using the formula: RR=(1-e^HR ln(1-r)^)/r, where r is the rate of outcome in reference group (37).

Studies reporting the effect size for each unit increase in lifestyle score were included in a separate meta-analysis. Given the heterogeneity across studies in study population characteristics and healthy lifestyle scoring (the number, component, and weights of different lifestyle factors), all meta-analyses were conducted using random-effects model.

Pre-specified subgroup analyses were performed to detect potential effect modification, according to study design (cohort, case-control), study setting (Europe, North America, Asia, Africa), ethnicity of the predominant study population (Caucasian, Asian, African, African American), mean age (<60, ≥60 years), follow-up time (<10 years, ≥10 years, unknown), gender (women, men, both), scoring system [simple lifestyle score, WCRF/AICR (World Cancer Research Fund and the American Institute for Cancer Research) recommendation adherence score, ACS (American Cancer Society) guideline adherence score], examined factors (five factors, smoking excluded, smoking and diet excluded, smoking, alcohol, and body measure excluded), and study quality (high, moderate, low). Cochran’s Q test and I^2^ were used to assess the heterogeneity across studies, with p<0.05 and/or I^2^>50% indicating significant heterogeneity (38, 39). Potential publication bias was assessed by visual inspection of funnel plots as well as the Egger’s test when the number of included studies is more than 10 (40). P-value <0.05 in Egger’s test indicates presence of publication bias. Sensitivity analysis was performed to assess the robustness of the summary estimates by excluding studies of low quality and by including studies with relative comprehensive covariates only.

Random-effect dose-response analysis with one-stage method was used to generate the study slope lines (41). To minimize the impact of methodological heterogeneity on effect estimates in dose-response analysis, we only included studies using simple unweighted scoring. We further standardized the score scale so that each point represents adherence to one healthy lifestyle. For example, we modified the score scale in studies that assigned two points to each lifestyle factors by multiplying the original score by 0.5. We investigated potential non-linear relationship by using restricted cubic splines with three knots located at 10^th^, 50^th^, and 90^th^ percentiles of the exposure category (42). These three knots accordingly represented 0.5, 2.5, and 4.5 points in the 5-point healthy lifestyle score scale. The curve segments before the first knot and after the last knot was assumed to be linear. Akaike information criteria (AIC) was used to compare the fitness of models, with the lower AIC indicating the better-fitting model (43). All quantitative data analyses were conducted by using Stata 14.0 (Stata Corp LP, College Station, TX, USA).

## Results

### Summary of Study Selection

**Figure 1:**
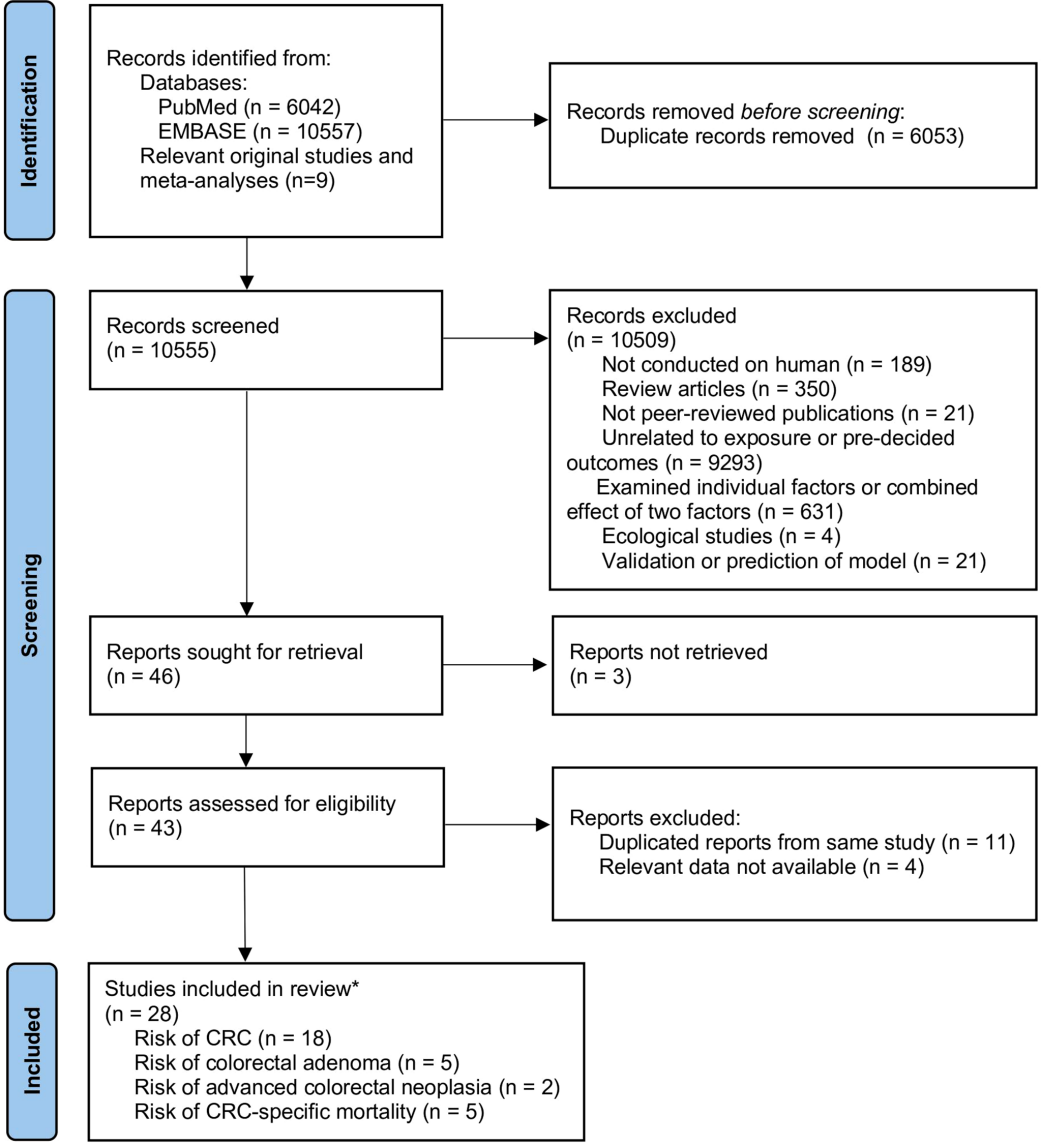
Flow chart of study selection.

A total of 10,555 unique records were identified from the literature search, 28 of which were considered eligible and were included. Among the eligible studies, 18 reported the risk of CRC (29–32, 34, 35, 44–55), five reported the risk of incident CRA (56–60), two reported the risk of advanced colorectal neoplasia (57, 61), and five reported CRC-specific mortality (33, 50, 62–64). The details of study selection are outlined in **Figure 1**. Among the five studies on CRC-specific mortality, two were conducted on CRC patients (50, 62) while the other three were conducted among healthy populations (33, 63, 64).

### Characteristics of Included Studies

The characteristics of included studies are shown in **Table 1** and **Supplementary Table 3**. Among the 28 studies included in the analyses, 18 were cohort studies, eight case-control studies, and two cross-sectional studies. The mean age at baseline ranged from 46.1 to 78.9 years. Eight studies were conducted among women (32, 35, 46, 47, 50, 54, 55, 62) while one was conducted among men (51); the other studies included both sexes, one of which (49) reported data separately for men and women. In terms of the study setting, 12 were conducted in the US, 10 in European countries, five in Asia, and one in Africa. The mean sample size was 51,735, with a range between 138 and 521,330. The median follow-up of cohort studies ranged from 3.1 years to >24 years.

**Table 1:** Basic characteristics of the included studies (n=28).

Study ID	Country	Study design	Sample size	Mean age (range)	Male %	Median follow-up year	Outcomes assessed (n)	Healthy lifestyle components					
								Diet	Smoking	Alcohol use	Physical activity	Body measure	Other
Aleksandrova (52)	10 European countries	Cohort	521330	51.8 (25-70)	35.0	12.0	CRC (3579) Colon cancer (2359) Rectal cancer (1390)	X	X	X	X	X	
Barrubes (53)	Spain	Cohort	7216	67.0 (62-72)	57.4	6.0	CRC (101)	X	X	X	X	X	
Byrd (56)	US	Case-control	2751	55.5 (NA)	47.5	NA	CRA (765)		X	X	X	X	
Carr (29)	Germany	Case-control	7124	68.2 (32-99)	60.8	NA	CRC (4092) Colon cancer (24579) Rectal cancer (1633)	X	X	X	X	X	
Cheng (54)	US	Cohort	35221	61.7 (55-69)	0	>10.0	CRC (1737)		X		X	X	
Cho (34)	South Korea	Case-control	1927	56.1 (NA)	68.3	NA	CRC (632) Colon cancer (318) Rectal cancer (304)	X	X	X	X	X	
Dartois (55)	France	Cohort	64732	NA (43-68)	0	15.0	CRC (481)	X	X	X	X	X	
Erdrich (35)	US	Cohort	81092	63.0 (40-89)	0	24.0	Colon cancer (1127)	X	X	X	X	X	
Erben (57)	Germany	Cross-sectional	13600	62.9 (NA)	50.3	NA	CRA (2839) Advanced colorectal neoplasia (1375)	X	X	X	X	X	
Fliss-Isakov (58)	Israel	Case-control	788	58.8 (NA)	52.7	NA	CRA (403)	X	X		X	X	
Fu (59)	US	Case-control	5208	57.4 (40-75)	63.0	NA	Advanced CRA (386) Non-advanced CRA (1220)	X	X			X	X ^e^
Hang (30)	China	Case-control	61693	68.9 (23-98)	45.2	NA	CRC (1144)	X		X	X		X ^a^
Harnack (32)	US	Cohort	34708	61.7 (55-69)	0	13.0	Colon cancer (619)	X		X	X	X	
Hastert (44)	US	Cohort	66920	61.1 (50-76)	49.0	7.6	CRC (546)	X		X	X	X	
Hatime (45)	Morocco	Case-control	2906	56.0 (NA)	49.3	NA	CRC (1453) Colon cancer (729) Rectal cancer (724)	X	X	X	X	X	
Inoue-Choi (62)	US	Cohort	2017	78.9 (72-88)	0	5.4	CRC-specific mortality (23)	X		X	X	X	
Jones (46)	UK	Cohort	30963	52.3 (NA)	0	18.7	CRC (444) Colon cancer (322) Rectal cancer (146)	X		X	X	X	X ^b^
Kirkegaard (31)	Denmark	Cohort	55487	56.0 (50-64)	48.0	9.9	CRC (678) Colon cancer (420) Rectal cancer (258)	X	X	X	X	X	
Knudsen (61)	Norway	Cross-sectional	6315	62.0 (NA)	48.0	NA	Advanced colorectal neoplasia (311)	X	X	X	X	X	
Lohse (63)	Switzerland	Cohort	16722	46.1 (25-74)	48.8	21.7 ^d^	CRC-specific mortality (79)	X		X	X	X	
Nomura (47)	US	Cohort	49103	38.2 (21-69)	0	15.1	CRC (328) Colon cancer (259)	X		X	X	X	
Odegaard (48)	Singapore	Cohort	50466	55.9 (45-74)	46.4	11.5	CRC (969) Colon cancer (590) Rectal cancer (379)	X	X	X	X	X	X ^a^
Petimar (49) (m)^c^	US	Cohort	45442	52.8 (40-75)	100	>24.0	CRC (1151) Colon cancer (907) Rectal cancer (244)	X		X	X	X	
Petimar (49) (f)^c^	US	Cohort	68977	52.8 (30-55)	0	>24.0	CRC (1298) Colon cancer (1023) Rectal cancer (275)	X		X	X	X	
Romaguera (33)	10 European countries	Cohort	3292	64.6 (NA)	45.5	4.2	CRC-specific mortality (872)	X		X	X	X	X^b^
Sotos-Prieto (64)	US	Cohort	87113	51.7 (40-75)	66.9	NA	CRC-specific mortality (684)	X	X	X	X	X	
Tabung (60)	US	Case-control	138	NA (30-80)	49.3	NA	CRA (47)	X	X	X	X	X	
Thomson (50)	US	Cohort	65838	63.2 (50-79)	0	12.6	CRC (751) CRC-specific mortality (190)	X		X	X	X	
Zhang (51)	China	Cohort	59503	55.3 (40-74)	100	9.3	CRC (674) Colon cancer (400) Rectal cancer (274)	X		X	X	X	

a Sleeping duration included as a component;

b Breastfeeding (applicable to women) included as a component;

c Petimar 2019 reported outcome on males and females separately and was therefore included as two separated studies in analysis;

d mean follow-up;

e Regular nonsteroidal anti-inflammatory drug use; CRC, colorectal cancer; CRA, colorectal adenoma; NA, not available.

### Quality Assessment

Using the Newcastle-Ottawa Scale, the included cohort studies received ratings ranging from five to eight stars. Nine studies were rated as of high quality (31, 32, 35, 48, 49, 51, 54, 62, 63), nine studies of moderate quality (33, 44, 46, 47, 50, 52, 53, 55, 64). None of the studies got star for the ascertainment of exposure because the lifestyle habits were self-reported by participants. In some studies, the sample was not well representative of general population. The included case-control studies received three to seven stars. Two studies were rated as high quality (29, 60), two moderate quality (30, 59), and four low quality (34, 45, 56, 58). The common biases in low quality studies were introduced by poor selection of cases and controls and unclear outcome ascertainment. Of the two cross-sectional studies included, one was rated as high quality (61) while the other moderate quality (57). The assessment results of all the included studies are described in **Supplementary Table 4**.

### Meta-Analysis

#### Overall Risk for CRC

**Figure 2:**
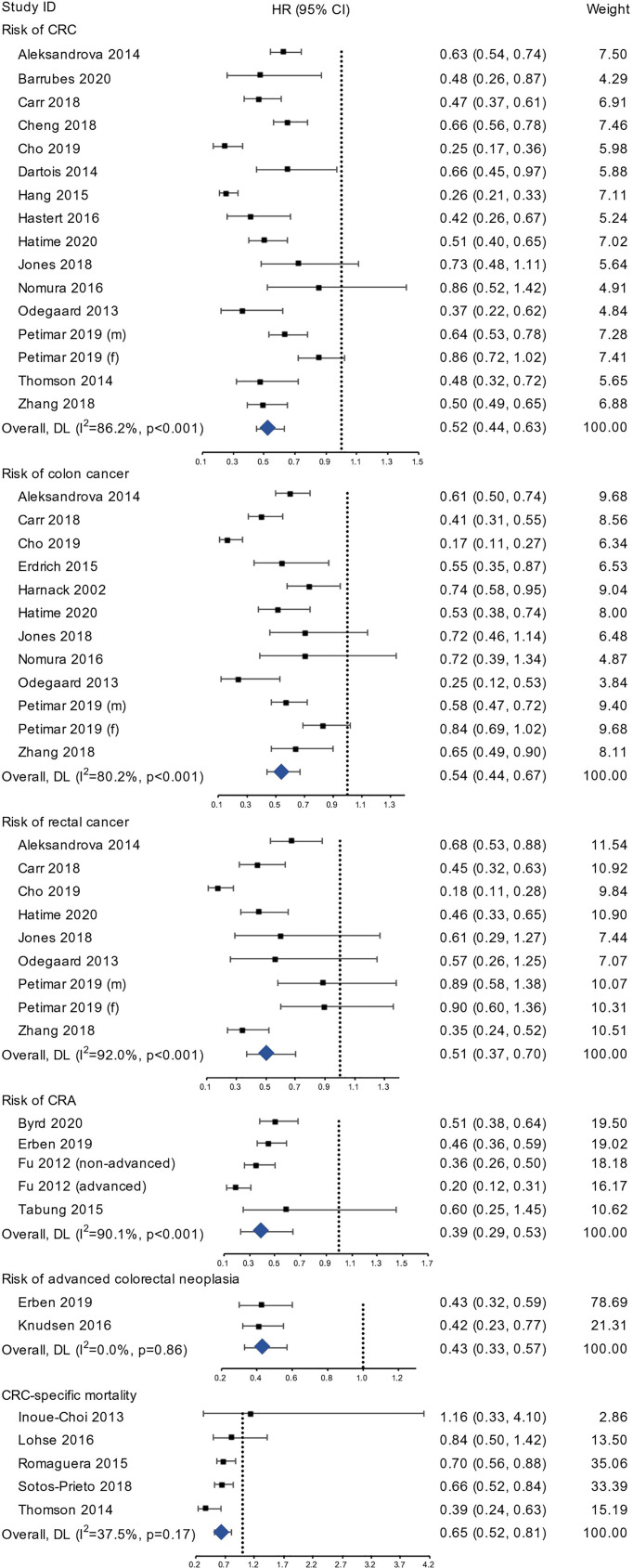
The forest plots of risk of CRC, colon cancer, rectal cancer, CRA, advanced colorectal neoplasia, and CRC-specific mortality.

We included 15 studies (1,139,361 participants), 11 studies (953,541 participants) and 8 studies (788,038 participants) in the meta-analyses of CRC, colon cancer and rectal cancer, respectively (**Figure 2**). Compared with the least healthy lifestyle, the adherence of the healthiest lifestyle was associated with 48% (HR=0.52, 95% CI 0.44-0.63, I^2 ^= 86.2%), 46% (HR=0.54, 95% CI 0.44-0.67, I^2 ^= 80.2%), and 49% (HR=0.51, 95% CI 0.37-0.70, I^2 ^= 92.0%) lower risk of CRC, colon cancer and rectal cancer, respectively. After pooling the studies using continuous lifestyle scores, the results showed that the per 1-unit increase in healthy lifestyle score was associated with a pooled HR of 0.88 (95% CI 0.84-0.92) for CRC, 0.87 (95% CI 0.83-0.92) for colon cancer, and 0.86 (95% CI 0.79-0.90) for rectal cancer (**Supplementary Figure 1**).

**Figure 3:**
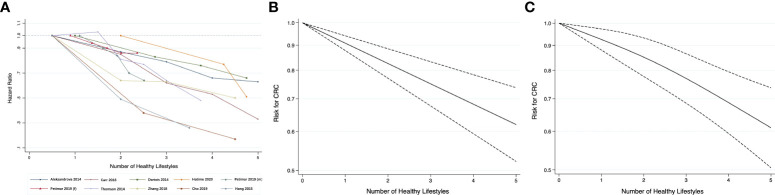
**(A)** Line graph of association between healthy lifestyles and risk for CRC; dose-response relationship between the number of healthy lifestyles and the risk for CRC: **(B)** Linear trend; **(C)** Restricted cubic splines.

Nine studies were included in the dose-response meta-analysis for risk of CRC (29, 30, 45, 49–52, 55, 65). The reported risk estimates for association between the number of present healthy lifestyles and risk of CRC from these studies generally showed an inverse linear relationship, as displayed in **Figure 3A**. The AIC was -54.7 for linear model (**Figure 3B**) and -44.1 for model using cubic splines (**Figure 3C**). Given the lower AIC, the linear model was considered better-fitting and was adopted for further analysis. Overall, the pooled HR for CRC was 0.91 (95% CI 0.88-0.94) per 1-unit increase in the number of healthy lifestyles, similar to the overall estimate in the meta-analysis of continuous lifestyle scores.

**Figure 4:**
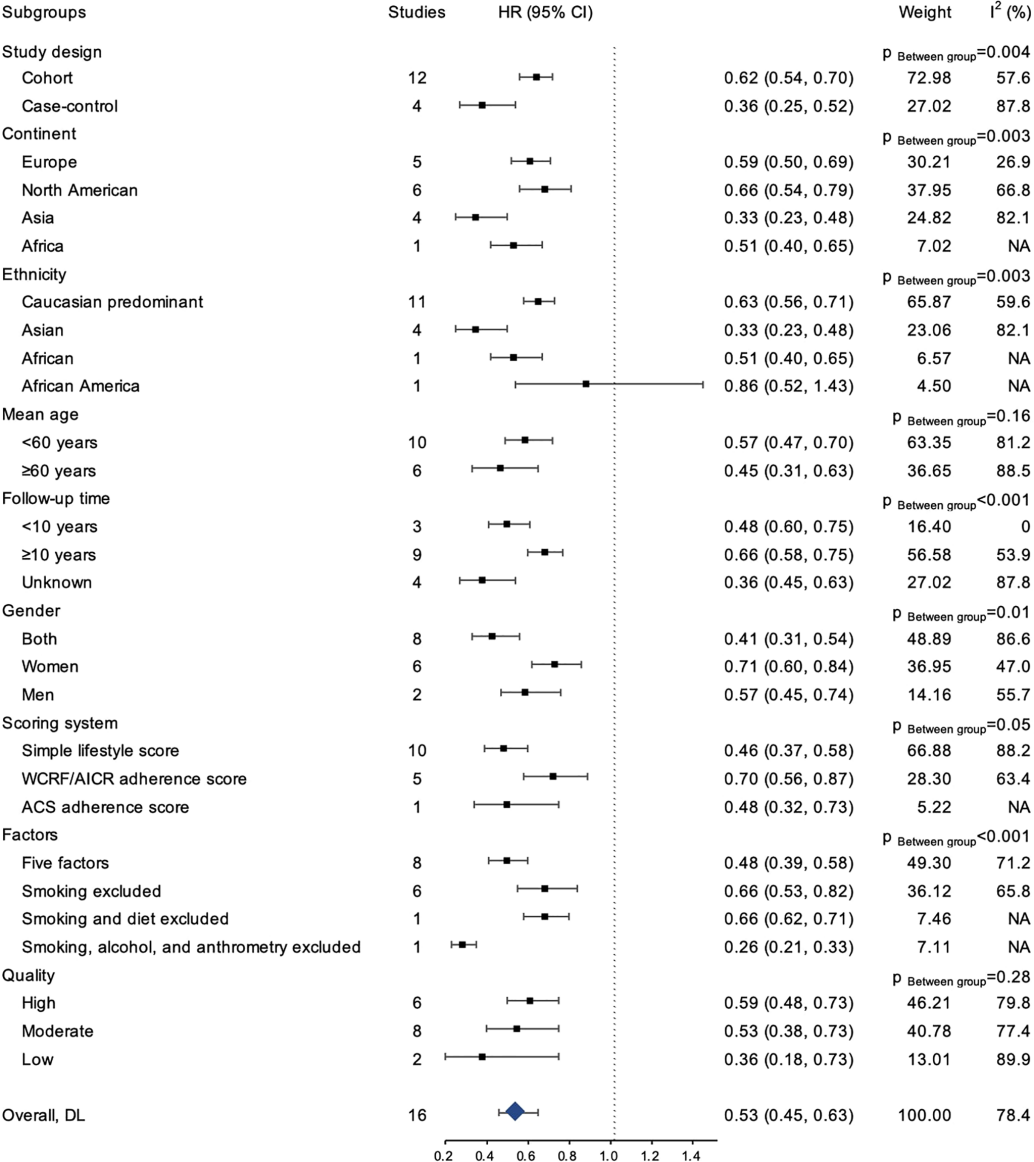
The results of the subgroup analyses for risk of CRC.

The result of subgroup analyses was presented in **Figure 4**. Overall, the inverse association between healthy lifestyle and risk for CRC was consistently observed within each subgroup (HR ranging from 0.26 to 0.86), and the association was statistically significant for all subgroups except among African Americans. Similarly, the inverse association for colon cancer was statistically significant in all subgroups, except among African Americans (**Supplementary Figure 2**). The associations for rectal cancer similarly remained directionally consistent with the primary analysis, although statistical significance was not reached in some subgroups (**Supplementary Figure 3**).

In sensitivity analysis, we conducted separate meta-analysis for risk of CRC of (1) all studies after excluding the two studies of low quality (34, 45); and (2) the three studies that adjusted for a relative comprehensive list of covariates (socio-demographic factors, family history, and intake of nutritional supplement and nonsteroidal anti-inflammatory drugs at baseline) (45, 49, 50). The results of these analyses were consistent with main analysis (**Supplementary Figure 4**).

#### Overall Risk for Colorectal Adenoma and Advanced Colorectal Neoplasia

Four studies (21,697 participants) reporting risk for incident colorectal adenoma using categorical lifestyle variables were included in the analysis (**Figure 2**), and the pooled HR was 0.39 (95% CI 0.29-0.53, I^2 ^= 90.1%). Two studies reported the risk for advanced colorectal neoplasia and the pooled HR for the healthiest group was 0.43 (95% CI 0.33-0.57, I^2 ^= 0.0%).

#### Overall Risk for CRC-Specific Mortality

Five studies with 174,982 participants were included in the analysis of CRC-specific mortality. The group with the highest lifestyle score showed 35% lower risk (HR=0.65, 95% CI 0.52-0.81, I^2 ^= 37.5%) compared to the group with lowest score (**Figure 2**). Using continuous lifestyle score, 1-unit increase in healthy lifestyle score was associated with an HR of 0.84 (95% CI 0.77-0.91) (**Supplementary Figure 1**). Subgroup analyses showed largely consistent results of similar directions (**Supplementary Figure 5**).

#### Publication Bias

The result of Egger’s test suggested no evidence of significant publication bias (p=0.23 for CRC risk, p=0.09 for colon cancer risk). The funnel plots for these two outcomes with more than 10 studies showed overall asymmetrical pattern (**Supplementary Figure 6**).

## Discussion

This systematic review and meta-analysis found that adopting multiple healthy lifestyles is associated with a considerably lower risk of multiple colorectal diseases. Compared with individuals with the least healthy lifestyle, those with the healthiest lifestyle had 48%, 46% and 49% lower risk of CRC, colon cancer, and rectal cancer, respectively. The associations were consistent across populations with different socio-demographic characteristics. A dose-response relationship between the number of healthy lifestyles and risk of CRC was identified, and adoption of each additional healthy lifestyle lowers the risk of CRC by 9% on average. We have also found that adherence to the healthiest lifestyle was associated with 61% lower risk of incident colorectal adenoma and 57% lower risk of advanced colorectal neoplasia. Among CRC survivors, those with the healthiest lifestyle had 31% lower risk of CRC-specific mortality.

The dose-response relationship between various individual lifestyle factors and CRC risk has been well established. It is reported that the relative risk for developing CRC is 0.90 for an increase of 10 g/day of dietary fibre (14), 1.24 for 120 g/day increase of red meat, 1.36 for 30 g/day increase of processed meat (66), 1.34 for one-point increase of Dietary Inflammatory Index (67), 1.38 for 50 g/day increase of alcohol intake (68), 1.07 for 2 kg/m^2^ increase in BMI, 1.04 for 2-cm increase in waist circumference (69), and 0.99 for 1 metabolic equivalent task (MET)-hour/week increase when the physical activity is over 10 MET-hour/week (70). In this study, we further revealed a dose-response association between the number of adopted healthy lifestyles and CRC risk, which further supports the significant difference in CRC risk between those with the healthiest lifestyle and those with the least. Previous studies have reported that healthy or unhealthy lifestyles tend to aggregate in individuals (71, 72), and the prevalence of adopting a healthiest lifestyle is generally low among general populations. For example, only 5.7% of the study population reported having all four healthy lifestyles (non-smoking, low alcohol consumption, sufficient fruit and vegetable consumption, regular physical activity) in England (73), while in Netherland, approximately 20% of the general population presented at least three of the five unhealthy lifestyles (smoking, low vegetable and fruit consumption, excessive alcohol intake, low physical activity) examined and all lifestyle factors showed significant clustering (72). It can, therefore, be expected that promotion of all healthy lifestyles among the populations could produce a synergistic effect on preventing CRC. A prospective study from the US estimated that 71% of colon cancer risk was attributable to a combination of unhealthy lifestyles, including being overweight, physical inactivity, alcohol consumption, smoking, and unhealthy diet (74). A prospective study in Denmark estimated that an overall 16% of the new CRC cases (22% for male and 11% for female) were attributable to lack of adherence to a combination of five healthy lifestyle factors (healthy weight, physical activity, non-smoking, limited alcohol consumption, healthy diet) (52).

The subgroup analyses showed that associations between multiple lifestyle factors and colorectal cancer risk were largely consistent across different age groups, sexes, geographic settings, and ethnicities. This suggests that the promoting healthy lifestyles could benefit populations universally regardless of their demographic characteristics. However, it should be noted that the association was found not statistically significant in the group of African American, but given that only one study was included in this group, future studies with bigger sample size are warranted to further explore the association among this ethnicity.

A previous meta-analysis concluded that adherence to at least four of the five healthy lifestyles examined (non-smoking, normal weight, healthy diet, moderate or lower alcohol consumption, and regular physical activity) could reduce all-cause mortality by 66% compared to those with no more than one healthy lifestyle (75). Our result suggested that adopting the healthiest lifestyle lowers CRC-specific mortality by 35%, and this protective effect was found significant among both CRC patients and healthy populations. This indicates that improving lifestyles could significantly benefit CRC survivors. Previous evidence has demonstrated that a variety of interventions are effective in improving awareness of CRC risk factors and facilitating adoption of healthy lifestyles among CRC patients after diagnosis, including telephone-delivered coaching (76), combined exercise and dietary advice (77), and education and behavioral change techniques (78). Such strategies could be considered as an integral part of CRC management to improve survival outcomes.

This study is the first systematic review and meta-analysis to reveal the dose-response relationship between the number of healthy lifestyles and CRC risk. Given the lack of large randomized controlled trials to examine the effect of adopting multiple healthy lifestyles on the risk of CRC and CRA as well as the survival outcomes of CRC patients, our study has provided high quality evidence by including a pooled sample of more than one million participants and generating results that are unlikely to be affected by publication bias. Our findings support the recommendations by the World Health Organization (79), American Cancer Society (80), and WCRF/AICF (81) on prevention and management of cancer. Adopting healthy lifestyles could not only prevent colorectal adenoma and CRC among the general population, but also improve clinical outcomes among CRC survivors. Nonetheless, international evidence has shown that population at risk of CRC generally demonstrated low awareness of lifestyle risk factors of CRC, particularly the effect of weight and physical activity (78, 82, 83). It would be strategic to provide information to increase awareness of lifestyle risk factors and promote interventions targeting behavior change among both healthy populations and CRC patients. Similar to our findings, previous meta-analyses have revealed that adopting multiple healthy lifestyles is associated with lower risk for cardiovascular disease (84), all-cause mortality (85), and type 2 diabetes (86), and such associations are generally found to be consistent among different populations. Hence, promoting healthy lifestyles could produce health benefits not only for CRC, but also for a variety of other health outcomes.

A few limitations should be noted when interpreting the study results. First, composition of healthy lifestyle and definitions of lifestyle factors varied considerably across studies, which may introduce heterogeneity to meta-analysis. We used random-effects model to minimize the effect of heterogeneity on the overall estimates. To explore the potential heterogeneity caused by this variation, we conducted subgroup analysis based on scoring system and factor composition. Although heterogeneity remained substantial within subgroups, the protective effect was still consistent within each group. For dose-response relationship, we only included studies using unweighted score system to exclude this attrition. Second, most original studies are from high-income, Western settings whose populations are comprised predominantly of Caucasians. Hence, more evidence from other populations, particularly Asian and African populations is needed. Third, only five studies have reported on CRC-specific mortality, which may restrict the power of performing stratified analyses. Fourth, socio-economic status is a key determinant for individual lifestyles (87–89), but few included studies have fully adjusted for all socio-economic factors. Other factors related to CRC risk, such as the use of certain pharmacological agents and nutritional supplements at baseline, were not collected and therefore not adjusted for in some studies. Despite this heterogeneity of covariate adjustment, the consistent finding from sensitivity analysis supports the robustness of the pooled estimate from the main analysis. Lastly, immortal time bias may exist in the original cohort studies assessing mortality given the possible time gap between study initiation and exposure assessment.

In conclusion, the number of healthy lifestyle attributes is inversely correlated with the risk of colorectal adenoma, cancer, and CRC-specific mortality. Lifestyle interventions could effectively reduce incidence of CRC. Future research may explore the effect of complex interventions targeting multiple lifestyle factors on prevention and management of CRC; randomized controlled trial is needed to provide high-quality evidence on the combined effect of healthy lifestyles and CRC risk.

## Data Availability Statement

The original contributions presented in the study are included in the article/**Supplementary Material**. Further inquiries can be directed to the corresponding author.

## Author Contributions

JY, QF, and YZ designed the research. JY and QF conducted literature search and performed data extraction and meta-analysis. JHK and YZ reviewed studies for inclusion. JY, QF, JHK, and YZ contributed to the interpretation of data. JY drafted the paper. QF, JHK, and YZ made substantial contribution to the critical revision and editing of the manuscript. All authors contributed to the article and approved the submission version.

## Conflict of Interest

The authors declare that the research was conducted in the absence of any commercial or financial relationships that could be construed as a potential conflict of interest.

## Publisher’s Note

All claims expressed in this article are solely those of the authors and do not necessarily represent those of their affiliated organizations, or those of the publisher, the editors and the reviewers. Any product that may be evaluated in this article, or claim that may be made by its manufacturer, is not guaranteed or endorsed by the publisher.
