# Supplementary material for: Combined Effect of Healthy Lifestyle Factors and Risks of Colorectal Adenoma, Colorectal Cancer, and Colorectal Cancer Mortality: Systematic Review and Meta-Analysis
Source: Front Oncol. 2022 Jul 22;12:827019. doi: 10.3389/fonc.2022.827019 (PMC9353059; doi:10.3389/fonc.2022.827019)
Supplement: Supplementary file 1 [file DataSheet_1.docx]

**Supplementary Figure 1.** The forest plots of risk of CRC, colon cancer, rectal cancer, and CRC-specific mortality using continuous exposure (1-unit increase)

**Supplementary Figure 2.** The results of the subgroup analyses for risk of colon cancer

*Five factors: diet, smoking, physical activity, alcohol consumption, arthrometry

**Supplementary Figure 3.** The results of the subgroup analyses for risk of rectal cancer

**Supplementary Figure 4.** Results of sensitivity analysis for risk of colorectal cancer: (A) meta-analysis of studies excluding those of low quality ; (B) meta-analysis of studies that adjusted for socio-demographic factors, family history, use of nutritional supplements (calcium and/or multivitamin) and nonsteroidal anti-inflammatory drugs at baseline.

*Five factors: diet, smoking, physical activity, alcohol consumption, arthrometry

**Supplementary Figure 5.** The results of the subgroup analyses for CRC-specific mortality.

**Supplementary Figure 6.** Funnel plots: (A) risk of CRC: Egger’s test p=0.23; (B) risk of colon cancer: Egger’s test p=0.09.

**Supplementary Table 1.** Literature search strategy for MEDLINE

| Search | Query |
| --- | --- |
| 1 | Search: ((((((((((combined[Title/Abstract]) OR (combination[Title/Abstract])) OR (multiple[Title/Abstract])) OR (joint effect*[Title/Abstract])) OR (integrated[Title/Abstract])) OR (merged effect*[Title/Abstract])) OR (adherence to[Title/Abstract])) OR (adhering to[Title/Abstract])) OR (index[Title/Abstract])) OR (score[Title/Abstract])) OR (compositional[Title/Abstract]) |
| 2 | Search: (((((((lifestyle*[Title/Abstract]) OR (modifi*[Title/Abstract])) OR (risk factor*[Title/Abstract])) OR (protective factor*[Title/Abstract])) OR (health* behaviour*[Title/Abstract])) OR (health* behavior*[Title/Abstract])) OR (behavioural factor*[Title/Abstract])) OR (behavioral factor*[Title/Abstract]) |
| 3 | Search: #1 AND #2 |
| 4 | Search: (((((colorectal[Title/Abstract]) OR (colon[Title/Abstract])) OR (colonic[Title/Abstract])) OR (sigmoid[Title/Abstract])) OR (rectal[Title/Abstract])) OR (rectum[Title/Abstract]) |
| 5 | Search: ((((((cancer[Title/Abstract]) OR (carcinoma[Title/Abstract])) OR (neoplasm[Title/Abstract])) OR (tumor[Title/Abstract])) OR (tumour[Title/Abstract])) OR (malignancy[Title/Abstract])) OR (adenoma[Title/Abstract]) |
| 6 | Search: #4 AND #5 |
| 7 | Search: #3 AND #6 |

**Supplementary Table 2.** Literature search strategy for EMBASE

| Search | Query |
| --- | --- |
| 1 | (combined or combination or multiple or joined effect* or integrated or merged effect* or adherence or adhering to or index or score or compositional).ti. or (combined or combination or multiple or joined effect* or integrated or merged effect* or adherence or adhering to or index or score or compositional).ab. |
| 2 | (lifestyle* or modifi* or risk factor* or protective factor* or health behaviour* of health behavior* or behavioural factor* or behavioral factor*).ti. or (lifestyle* or modifi* or risk factor* or protective factor* or health behaviour* of health behavior* or behavioural factor* or behavioral factor*).ab. |
| 3 | 1 and 2 |
| 4 | (colorectal or colon or colonic or sigmoid or rectal or rectum).ti. or (colorectal or colon or colonic or sigmoid or rectal or rectum).ab. |
| 5 | (cancer or carcinoma or neoplasm or tumour or tumor or malignancy or adenoma).ti. or cancer or carcinoma or neoplasm or tumour or tumor or malignancy or adenoma).ab. |
| 6 | 4 and 5 |
| 7 | 3 and 6 |

**Supplementary Table 3.** Basic characteristics of the included studies (n=28).

| *First author, year* | *Study of data source* | *Setting* | *Study design*  *(Sample size)* | *Median follow-up year* | *Male %* | *Mean age (range)* | *No. of outcomes* | *Definition of healthy lifestyle* | *Group comparison* | *Covariates adjusted for* |
| --- | --- | --- | --- | --- | --- | --- | --- | --- | --- | --- |
| Aleksandrova 2014 | European Prospective Investigation into Cancer and Nutrition (EPIC) cohort | 10 European countries | Cohort  (N=521330) | 12.0 | 35.0 | 51.8 (25-70) | 3759 CRC  2369 Colon cancer  1390 Rectal cancer | Diet (Dietary quality index of colorectal cancer related food): 0. 0-4 points; 1. 5-8 points.  Smoking: 0. current smoker; 1. never or former smokers.  Alcohol: 0. two standard drinks a day (>24 g/d) for men and one standard drink a day (>12 g/d) for women; 1. two standard drinks a day (≤24 g/d) for men and one standard drink a day (≤12 g/d) for women.  PA: 0. sedentary or standing occupation and recreational METs ≤57 for men and METs ≤82 for women; 1. manual or heavy manual occupation and recreational METs >57 for men and METs >82 for women.  Arthrometry: 0. BMI ≥25 kg/m^2^ or waist circumference ≥94 cm for men and ≥80 cm for women; 1. BMI 18 to 25 kg/m^2^ or waist circumference <94 for men cm and <80 for women. | 0-1 points  2 points  3 points  4 points  5 points | Age, sex, and education. |
| Barrubes 2020 | Spanish PREvencion con DIeta MEDiterranea (PREDIMED) cohort | Spain | Cohort  (N=7216) | 6.0 | 57.4 | 67.0 (62-72) | 101 CRC | Diet (Alternate Healthy Eating Index): 0. lower 60%; 1. upper 40%.  Smoking: 0. current or former smokers; 1. never smokers.  Alcohol: 0. < 5g/d or >15 g/d for women and >30 g/d for men; 1.5 to 15 g/d for women and 5 to 30 g/d for men.  PA: 0. <30 min/d MVPA; 1. ≥30 min/d MVPA.  BMI: 0. <18.5 or ≥25 kg/m^2^; 1. 18.5 to 24.9 kg/m^2^. | Tertile 1  Tertile 2  Tertile 3  Incremental (per 1-point increase) | Age, sex, dietary intervention, family history of cancer, education, history of diabetes, baseline energy intake, and treatment with aspirin at baseline. |
| Byrd 2020 |  | US | Case-control  (N=2751) | NA | 47.5 | 55.5 (NA) | 765 CRA | *Beta coefficient weights:*  Alcohol: 0.3: >7 drinks/w (women) or >14 drinks/w (men) vs. non-drinker; -0.66: 1-7 drinks/w (women) or 1-14 drinks/w (men) vs. non-drinker.  PA: -0.18: mid tertile of MET-h/w; -0.41: highest tertile of MET-h/w.  Smoking: 0.50: current smokers vs. current non-smokers.  BMI: 0.89: 25-29.9 kg/m^2^ vs. <24.99 kg/m^2^; 1.57: ≥30 kg/m^2^ vs. >24.99 kg/m^2^. | Quintile 1  Quintile 2  Quintile 3  Quintile 4  Quintile 5 | Age, sex, regular aspirin or other NSAID use, hormone therapy use (women only), family history of CRC in a ﬁrst-degree relative, former smoking status, total energy intake, study of cohort, and the equally-weighted Diet Inflammation Score. |
| Carr 2018 | DACHS study | Germany | Case-control  (N= 7124) | NA | 60.8 | 68.2 (32-99) | 4092 CRC  2459 Colon cancer  1633 Rectal cancer | Diet (23-item FFQ): 0. lower 60% (0-33 points); 1. upper 40% (≥34 points).  Smoking: 0. never smokers and former smokers. 1. current smokers.  PA: 0.>500 MET minutes/w 1. ≥500 MET minutes/w.  BMI: 0. ≤18.5 or ≥25 kg/m^2^; 1. >18.5 to <25 kg/m^2^. | 0-1 point  2 points  3 points  4 points  5 points | Age, sex, education, family history of CRC, history of colonoscopy, participation in a health checkup, ever regular use of NSAIDs. |
| Cheng 2018 | Iowa Women's Health Study | US | Cohort  (N=35221) | >10.0 | 0 | 61.7 (55-69) | 1737 CRC | Smoking: 1. current smoker; 3. former smoker; 5. never smoker.  PA: 1. no VPA or MPA <twice/w; 3. VPA ≥twice/w or MPA >4 times/w; 5.VPA once/w plus MPA once/w, or MPA 2–4 times/w.  BMI: 1. ≥30.0 kg/m^2^; 3. 25.0–29.9 kg/m^2^; 5. <25.0 kg/m^2^. | Quintile 1  Quintile 2  Quintile 3  Quintile 4  Quintile 5 | Age, family history of CRC in a ﬁrst-degree relative, education, total energy intake, arthritis, use of hormone replacement therapy, and evolutionary-concordant diet score. |
| Cho 2019 | - | South Korea | Case-control  (N=1927) | NA | 68.3 | 56.1 (NA) | 632 CRC  318 Colon cancer  304 Rectal cancer | Diet (Dietary inflammatory index, DII): 0. ≤ median DII; 1. >median DII.  Smoking: 0. never smokers; 1. ever smokers.  Alcohol:0. ≥30 g/d; 1. ≤30 g/d.  PA: 0. Conducting regular physical exercise; 1. no regular physical exercise.  BMI: 0. <25 kg/m^2^; 1. ≥25 kg/m^2^. | 0-1 point  2-3 points  4 points  5 points | Age, sex, family history of CRC, education; if applicable, prior BMI, physical activity, smoking, alcohol, and dietary inflammatory index. |
| Dartois 2014 | French E3N | France | Cohort  (N=64732) | 15.0 | 0 | NA (43-68) | 481 CRC | Fruit and vegetable consumption: 0. <3.5 servings/day; 0.5. ≥3.5 and <5 servings/day; 1. ≥5 servings/day.  Smoking: 0. current smokers; 0.5. former smokers; 1. never smokers.  Alcohol: 0. ≥2 drinks/day; 0.5. ≥1 and <2 drinks/day; 1. ≤1 drinks/day.  PA: 0. <10 MET-h/w; 0.5. ≥10 and <20 MET-h/w; 1. ≥20 MET-h/w.  BMI: 0. <16 or ≥30 kg/m^2^; 0.5. ≥16 and <18.5 kg/m^2^or ≥25 and <30 kg/m^2^; 1. ≥18.5 and <25 kg/m^2^. | 0-2 points  2.5-3 points  3.5-4 points  4.5-5 points | Education, residence, ﬁrst-degree family history of any cancer, professional activity, use of oral contraceptives, age at menarche and number of children, age at ﬁrst full-term pregnancy, menopausal status, and use of menopausal hormone therapy. |
| Erdrich 2015 | Nurses’ Health Study | US | Cohort  (N=81092) | 24.0 | 0 | 63.0 (40-89) | 1127 Colon cancer | Multivitamin use: 0. current use ≥15 years; 1. none, past, or current use <15 years.  Calcium intake: 0. ≥700 mg/d; 1. <700 mg/d.  Smoking: 0. <10 pack-year before age 30; 1. ≥10 pack-year before age 30.  Alcohol: 0. <30 g/d; 1. ≥30 g/d.  PA: 0. >21 h/w; 1. ≤21 h/w.  BMI: 0. <25 kg/m^2^; 1. ≥25 kg/m^2^. | 0-1 points  2 points  3 points  4 points  5 points | Age, history of CRC in a parent or sibling, history of colonoscopy/sigmoidoscopy, regular aspirin use, postmenopausal hormone use. |
| Erben 2019 | CRA  Advanced colorectal neoplasia | Germany | Cross-sectional  (N=13600) | NA | 50.3 | 62.9 (NA) | 2839 CRA  1375 advanced  colorectal neoplasia | Diet (FFQ score): 0. >34 points; 1. ≥34 points.  Smoking: 0. current smokers or former smokers (≥30 pack years); 1. never smokers or former smokers (<30 pack years).  Alcohol: 0. >24g/d (men), >12g/d (women); 1. ≤24g/d (men), ≤12g/d (women).  PA: 0. <50 minutes of moderate-intensity or 75 minutes of vigorous-intensity aerobic physical activity throughout the week or an equivalent combination of moderate and vigorous intensity physical activity; 1. ≥50 minutes of moderate-intensity or 75 minutes of vigorous-intensity aerobic physical activity throughout the week or an equivalent combination of moderate and vigorous intensity physical activity.  BMI: 0. ≥25 kg/m^2^; 1. >18.5 and <25 kg/m^2^. | 0-1 point  2 points  3 pints  4 points  5 points | Age, sex, education, family history of CRC, history of large bowel endoscopy, participation in a health check-up, ever regular use of NSAIDs. |
| Fliss-Isakov 2020 |  | Israel | Case-control  (N=788) | NA | 52.7 | 58.8 (NA) | 403 CRA | Diet (Healthy diet score): 0. <5 points; 1. ≥5 points.  Smoking: 0. current or former smokers; 1. never smokers.  PA: 0. regular performance of strenuous physical activity < once/w or MPA < 5 times/w; 1. regular performance of strenuous physical activity ≥ once/w or MPA ≥5 times/w.  BMI: 0. ≥30 kg/m^2^; 1. <30 kg/m^2^. | Incremental increase (per 1-unit increase) | Age, history of CRC in a parent or sibling, history of colonoscopy/sigmoidoscopy, regular aspirin use, postmenopausal hormone use. |
| Fu 2012 | Tennessee Colorectal Polyp Study | US | Case-control  (N=5208) | NA | 63.0 | 57.4 (40-75) | 1444 CRA | Dietary calcium: 0. ≥ 971.4 mg/d; 1. <971.4 mg/d.  Dietary fiber intake: 0. ≥ 19.9 g/d; 1. <19.9 g/d.  Red meat intake: 0. <23.0 g/d; 1. ≥ 23.0 g/d.  Smoking: 0. never smokers; 1. ever or current smokers.  BMI: 0. <30 kg/m^2^; 1. ≥ 30 kg/m^2^.  Regular nonsteroidal anti-inflammatory drug use: 0. ever or current;  1. never. | 0-1 points  2 points  3 points  4 points  5-6 points | Age, sex, race, study site, education, indications for colonoscopy, total energy intake, recruitment before or after colonoscopy, and year of recruitment. |
| Hang 2015 | - | China | Case-control  (N=61693) | NA | 45.2 | 68.9 (23-98) | 1144 CRC | Red meat: 0. ≥3 days/w; 1. <3 days/w.  Vegetable: 0. <300 g/d; 1. ≥ 300 g/d.  PA (exercise out): 0. <3 times/w; 1. ≥3 times/w.  Sleep (duration): ≥8 h/d; <8 h/d. | 0-1 points  2 points  3-4 points | Age, sex, BMI, education, and family history of CRC. |
| Harnack 2002 | IWHS | US | Cohort  (N=34708) | 13.0 | 0 | 61.7 (55-69) | 619 Colon cancer | Grain: 0. <6 servings/d; 0.4. ≥6 servings/d.  Vegetable: 0. <3 servings/d; 0.4. ≥3 servings/d.  Fruit: 0. <2 servings/d; 0.4. ≥2 servings/d.  Milk: 0. <2 servings/d; 0.4. ≥2 servings/d.  Meat: 0. <2 servings/d; 0.4. ≥2 servings/d.  Variety of fruit: 0. ≤4 different items regularly; 0.5. 5-6 different items regularly; 1. ≥7 different items regularly.  Variety of vegetables: 0. ≤6 different items regularly; 0.5. 7-9 different items regularly; 1. ≥10 different items regularly.  Total fat: 0. >30% kcal from fat; 0.67. ≤30% kcal from fat.  Saturated fat: 0. >10% kcal from saturated fat; 0.67. ≤10% kcal from saturated fat.  Cholesterol: 0. >300mg daily; 0.67. ≤300mg daily.  Sweets and sugar-sweetened beverages: 0. ≥4 servings/d; 1. 2-3 servings/d;2. 0-1 servings/d.  Sodium intake: 0. >2400mg Na/d; 2. ≤2400mg Na/d.  PA: 0. <2 times/w; 1. 2-4 times/w; 2. >4 times/w.  Alcohol: 0. >1 drink/d; 2. ≤1 drink/d.  BMI: 0. ≥30 kg/m^2^; 1. 25.0-29.9 kg/m^2^; 2. <25.0 kg/m^2^. | Quantile 1  Quantile 2  Quantile 3  Quantile 4  Quantile 5 | Age, energy intake, smoking status, pack-years of cigarette smoking, hormone replacement therapy, and height. |
| Hastert 2016 | The VITamins And Lifestyle (VITAL) study | US | Cohort  (N=66920) | 7.6 | 49.0 | 61.1 (50-76) | 546 CRC | Energy density: 0. ≥125 kcal/100g or SSB ≥1 /w or fruit juice >3 servings/w; 1. <125 kcal/100g and SSB <1 /w and fruit juice ≤3 servings/w.  Plant food: 1. fruits and vegetables ≥5 servings/d and whole grains/legumes ≥1 serving/d; 0. fruits and vegetables <5 servings/d or whole grains/legumes <1 serving/d.  Red meat: 1. <18 oz/w and processed meat ≤1 serving/w; 0. ≥18 oz/w or processed meat >1 serving/w.  Alcohol: 0. >2/1 drink/d; 1. ≤ 2/1 drink/d.  PA (MPA): 1. ≥30 min/d and ≥5 d/w in at least 7 of the past 10 years; 0. <30 min/d or <5 d/w or <7 of the previous 10 years.  BMI: 0. <18.5 or ≥25 kg/m^2^; 1. ≥18.5 kg/m^2^ and <25 kg/m^2^. | 0 point  1 point  2 points  3 points  4-6 points | Age, sex, education, race/ethnicity, receipt of colonoscopy or sigmoidoscopy in 10 years before baseline, family history of colon or rectal cancer, NSAIDs use, history of cancer other than colorectal cancer, and total energy intake. |
| Hatime 2020 | - | Morocco | Case-control  (N=2906) | NA | 49.3 | 56.0 (NA) | 1453 CRC  729 Colon cancer  724 Rectal cancer | Fruit and vegetables: 0. 280–400 g/d; 1. ≥400 g/d.  Smoking: 0. current smoker; 0.5. ex-smoker; 1. never smoker.  Alcohol: 0. ≥20g/d; 0.5. ≥10 and <20 g/d; 1. <10 g/d.  PA: 0. <10 MET-h/w; 0.5. 10-20 MET-h/w; 1. >20 MET-h/w.  BMI: 0. <16 or ≥30 kg/m^2^; 0.5. 16-18.5 or 25-30 kg/m^2^; 1. 18.5-25 kg/m^2^. | Tertile 1  (<4 points)  Tertile 2  (4-4.5 points)  Tertile 3  (>4.5 points) | Age, residence, education, monthly income, family history of CRC; NSAIDs use; intake of red and processed meat; fiber, calcium, and total energy intake. |
| Inoue-Choi 2013 | Iowa Women’s Health Study | US | Cohort  (N=2017) | 5.4 | 0 | 78.9 (72-88) | 23 CRC-specific  mortality | Sugary drinks: 0. ≥250 g/d; 0.5. <250 g/d; 1. 0 g/d.  Dietary fibre: 0. <12.5 g/d.; 0.5. 13.5–24.9 g/d ; 0.5. ≥35 g/d.  Fruit and vegetable: 0. <3 FV servings/d; 0.5. 3 to <5 servings/d;1. ≥5 FV servings/d.  Red meat and processed meat: 0. ≥500 g/d or ≥50 g/d; 0.5. <500 g/d and 3 to <50 g/d; 1. <500 g/d and <3 g/d.  Sodium: 0. >2.4 g/d; 0.5. 1.6–2.4 g/d; 1. ≤1.5 g/d.  Alcohol: 0. >20 g/d; 0.5. 10–20.0 g/d; 1. ≤10 g/d.  PA: 0. <2 times/w vigorous or <5 times/w moderate activities; 0.5. ≥2 times/w vigorous or ≥5 times/w moderate activities; 1. 2-4 times/w moderate or once/w vigorous and moderate activities.  BMI: 0. ≥30.0 kg/m^2^; 0.5.18.5 to <25 kg/m^2^; 1. 18.5 to <25 kg/m^2^.  Breast feeding: 0. no; 0.5 0.1–5.9 months; 1. ≥6 months. | Quartile1  (1.5-4 points)  Quartile 2  (4.5 points)  Quartile 3  (5.0-5.5 points)  Quartile 4 (6.0-8.0 points) | Age, total number of comorbid conditions (accumulated, 1986–2004), perceived general health and current smoking, cancer stage, cancer type, cancer treatment, subsequent cancer diagnosis before 2004, current cancer treatment, and person-years since cancer diagnosis. |
| Jones 2018 | The UK Women’s Cohort Study (UKWCS) | UK | Cohort  (N=30963) | 18.7 | 0 | 52.3 (NA) | 444 CRC  322 Colon cancer  146 Rectal cancer | Energy density: 0. >175 kcal/100g/d; 0.25 126–175 kcal/100g/d; 0.5. ≤125 kcal/100g/d.  Sugary drinks: 0. >250 g/d; 0.25. ≤250 g/d; 0.5. 0 g/d.  Fruit and vegetable: 0. <200 g/d; 0.25. 200–399 g/d; 0.5. ≥400 g/d.  Dietary fibre: 0. <12.5 g/d.; 0.25. 12.5–24.9 g/d ; 0.5. ≥25 g/d.  Red meat and processed meat: 0. ≥500 g/d or ≥50 g/d; 0.5. <500 g/d and 3–49 g/d; 1. <500 g/d and <3 g/d.  Sodium: 0. >2.4 g/d; 0.5. 1.6–2.4 g/d; 1. ≤1.5 g/d.  Alcohol: 0. >20 g/d; 0.5. 10.1–20.0 g/d; 1. ≤10 g/d.  PA: 0. <15 min/d; 0.5. 15–30 min/d; 1. >30 min/d.  BMI: 0. <18.5 or ≥30.0 kg/m^2^; 0.5 25.0–29.9 kg/m^2^; 1. 18.5–24.9 kg/m^2^;  Breast feeding: 0. no; 0.5 0.1–5.9 months; 1. ≥6 months. | ≤3 points  >3, ≤5 points  >5, ≤8 points | Age, smoking status, socio-economic status, and family history of CRC. |
| Kirkegaard 2010 | Diet, Cancer and Health Cohort Study | Denmark | Cohort  (N=55487) | 9.9 | 48.0 | 56.0 (50-64) | 678 CRC  420 Colon cancer  258 Rectal cancer | Diet: 0. <600 g fruit and vegetables/day, < 500 g of red and processed meat/w, < 3 g dietary fibre per MJ of dietary energy,  or <30% of the total energy from fat; 1. ≥ 600 g fruit and vegetables/day, ≤ 500 g of red and processed meat/w, ≥ 3 g dietary fibre per MJ of dietary energy, and ≤ 30% of the total energy from fat.  Smoking: 0. current or former smokers; 1. never smokers.  Alcohol: 0. >7 drinks/w for women; >14 drinks/w for men 1. ≤ 7 drinks/w for women; ≤14 drinks/w for men.  PA: 0. <30 mins/d; 1. ≥30 mins/d or had a job with light manual activity (such as postal delivery) or heavy manual activity (such as forestry).  Waist circumference: 0. ≥88cm (women), ≥102cm (men); 1. <88 cm (women), <102 cm (men). | Incremental (per 1-point increase) | Education, use of NSAIDs, use of hormone replacement therapy (women only), and history of cancer in first degree relatives. |
| Knudsen 2016 |  | Norway | Cross-sectional  (N=6315) | NA | 48.0 | 62.0 (NA) | 311 Advanced  colorectal  neoplasia | Fruits and vegetables: 0. <3 servings/d; 0.5. ≥3 servings/d.  Fatty fish: 0. <1 serving/w; 0.5. ≥1 serving/w.  Red and processed meat: 0. >4 servings/w; 1. ≤4 servings/w.  Smoking: 0. current smokers; 1. non-smokers.  Alcohol: 0. >14 glasses/w (men) or >7 glasses/w (women); 1. ≤14 (men) or ≤7 (women).  PA (30 minutes): 0. <7 times/w; 1. ≥7 times/w.  BMI: 0. ≥25 kg/m^2^; 1. >25 kg/m^2^. | 0-1 points  2 points  3 points  4 points  5-6 points | Age, screenings arm, gender, study center, education, and whole meal bread. |
| Lohse 2016 | MONICA/NRP1A | Switzerland | Cohort  (N=16722) | 21.7 ^d^ | 48.8 | 46.1 (25-74) | 79 CRC-specific  mortality | Energy density (Energy density sub-score): 0. 0 point; 0.5. 1 point; 1. 2 or 3 points.  Fruits and vegetables (Intake the previous day): 0. neither; 0.5. either; 1. both.  Grains (Intake the previous day): 0. no; 1. yes.  Processed meat (Intake the previous day): 0. sausage product; 0.5. meat; 1. none.  Alcohol (Intake the previous day): 0. yes; 1. no.  PA: 0. <1 d/w; 0.5. 1 d/w; 1. ≥2 d/w.  Sedentary behaviour: 0. mostly sitting; 0.5. walking, cycling, other regular activities; 1. Regular exercise (MONICA cohort). 0. sedentary; 0.5. average; 1. exhausting. (NRP1A cohort)  BMI: 0. <18.5 or ≥30 kg/m^2^; 0.5. 25–29.9 kg/m^2^; 1. 18.5–24.9 kg/m^2^. | 0-3.5 points  4-4.5 points  5-9 points | Education, marital status, study, language region, nationality, and smoking status. |
| Nomura 2016 | Black Women’s Health Study (BWHS) | US | Cohort  (N=49103) | 15.1 | 0 | 38.2 (21-69) | 328 CRC  259 Colon cancer | Sugary drinks: 0. ≥250 g/d; 0.5. <250 g/d; 1. 0 g/d.  Fruit and vegetable and fiber: 0. <3 FV servings/d and/or <12.5 fiber g/w; 0.5. ≥3 and <5 FV servings/d and/or 12.5-<25 fiber g/w; 1. ≥5 FV servings/d and ≥25 g ﬁber/w;  Red meat and processed meat: 0. ≥500 g/w or ≥50 g/w; 0.5. <500 g/w and >3 and <50 g/w; 1. <500 g/w and <3 g/w;  Sodium: 0. >2.4 g/d. 0.5. >1.5 and ≤2.4 g/d; 1. ≤1.5 g/d.  Alcohol: 0. ≥14 servings/w (1 serving=10g) 0.5. 7-13 servings/w; 1. <7 servings/w.  PA: 0. <1 h/w vigorous or walking for exercise; 0.5. ≥8 h/d sitting or 1–2 h/w vigorous or 1–4 h/w walking for exercise 1. ≥3–4 h/w vigorous or ≥5–6 h/w walking for exercise and <8h/d sitting.  BMI and weight change in adulthood: 0. >30 kg/m^2^ and/or ≥13.62 kg; 0.5. >25, <30 kg/m^2^ and/or +6.81–13.61 kg; 1. ≥18.5, <25 kg/m^2^ and ≤+6.80 kg. | <3 points  3-4 points  >4 points | Age, geographic region of residence, caloric intake, smoking, family history of CRC, education, menopausal status, diabetes, insulin usage, aspirin usage, colonoscopy, sigmoidoscopy. |
| Odegaard 2013 | Singapore Chinese Health Study | Singapore | Cohort  (N=50466) | 11.5 | 46.4 | 55.9 (45-74) | 969 CRC  590 Colon cancer  379 Rectal cancer | Dietary pattern score: 0. lowest 25^th^ percentile; 1. middle 50^th^ percentile; 1. highest 25^th^ percentile.  Smoking: 0. heavy smokers (started to smoke before 15 years of age and smoked 13 or more cigarettes/d); 1. light smokers; 2. never smokers.  Alcohol: 0. >14 drinks/w; 1. 8-14 drinks/d; 2. 0-7 drinks/d.  PA: 0. <1.5 MET-h/w; 2. ≥ 1.5 MET-h/w.  BMI: 0. <18.5 or ≥27.5 kg/m^2^, 1. 18.5–27.4 kg/m^2^.  Sleep: 0. <6 or ≥9 h/d; 1. 6-8 h/d. | 0-3 points  4 points  5 points  6 points  7 points  8 points  9-10 points | Age, sex, year of enrollment, dialect, education, diabetes status, familial history of CRC, and energy intake. |
| Petimar 2019 (m) ^c^ | Nurses' Health Study  Health; Professionals Follow-up Study | US | Cohort  (N=100) | >24.0 |  | 52.8 (40-75) | 1151 CRC  907 Colon cancer  244 Rectal cancer | Fruits and vegetables: 0. <2.5 servings/d; 0.071. 2.5–4.9 servings/d; 0.143. ≥5 servings/d.  Fiber: 0. <15 g/d; 0.071. 15–29 g/d; 0.143. ≥30 g/d.  Whole grains or pulses: 0. <1.5 servings/d; 0.071. 1.5–2.9 servings/d; 0.143. ≥3 servings/d.  'Fast food' and other processed foods (refined grains, pastries, sweets, and salty snacks): 0. ≥3 servings/d; 0.071. 1.5–2.9 servings/d; 0.143. <1.5 servings/d.  Red meat: 0. ≥6 servings/w; 0.004. 3.1–5.9 servings/w; 0.071. ≤3 servings/w.  Processed meat: 0. ≥27 g/d; 0.004. 3–26 g/d; 0.071. <3 g/d.  Sodas and other beverages with added sugars: 0. ≥1 drink/d; 0.004. 0.1–0.9 drinks/d; 0.071. 0 drinks/d.  Juices: 0. ≥2 drinks/d; 0.004. 1–1.9 drinks/d; 0.071. <1 drink/d.  Alcohol: 0. ≥2/1 drinks/d; 0.071. 0.1–1.9/0.9 drinks/d; 0.143. 0 drinks/d.  PA: 0. <75 min/w; 0.25. 75–149 min/w; 0.5. ≥150 min/w.  TV watching: 0. ≥20 h/w; 0.25. 5–19.9 h/w; 0.5. <5 h/w.  BMI: 0. <15 or ≥30 kg/m^2^; 0.167. 15–18.4 or 25–29.9 kg/m^2^. 0.333. 18.5–24.9 kg/m^2^.  Weight gain in last 10 years: 0. ≥10 lb; 0.167. 0–9.9 lb; 0.333. no.  Waist circumference: 0. ≥40.2/34.6 inches; 0.167. 37–40.1/31.5–34.5 inches; 0.333. <37/31.5 inches. | Quantile 1  Quantile 2  Quantile 3  Quantile 4  Quantile 5 | Total energy intake, NSAID/aspirin use, family history of CRC, previous CRC screening via colonoscopy/ sigmoidoscopy, history of polyps, smoking, multivitamin use, supplemental calcium intake, young adult body mass index, menopausal status (women only), and postmenopausal hormone use (women only). |
| Petimar 2019 (f) ^c^ | Nurses' Health Study  Health; Professionals Follow-up Study | US | Cohort  (N=68977) | >24.0 | 0 | 52.8 (30-55) | 1298 CRC  1023 Colon cancer  275 Rectal cancer | Fruits and vegetables: 0. <2.5 servings/d; 0.071. 2.5–4.9 servings/d; 0.143. ≥5 servings/d.  Fiber: 0. <15 g/d; 0.071. 15–29 g/d; 0.143. ≥30 g/d.  Whole grains or pulses: 0. <1.5 servings/d; 0.071. 1.5–2.9 servings/d; 0.143. ≥3 servings/d.  'Fast food' and other processed foods (refined grains, pastries, sweets, and salty snacks): 0. ≥3 servings/d; 0.071. 1.5–2.9 servings/d; 0.143. <1.5 servings/d.  Red meat: 0. ≥6 servings/w; 0.004. 3.1–5.9 servings/w; 0.071. ≤3 servings/w.  Processed meat: 0. ≥27 g/d; 0.004. 3–26 g/d; 0.071. <3 g/d.  Sodas and other beverages with added sugars: 0. ≥1 drink/d; 0.004. 0.1–0.9 drinks/d; 0.071. 0 drinks/d.  Juices: 0. ≥2 drinks/d; 0.004. 1–1.9 drinks/d; 0.071. <1 drink/d.  Alcohol: 0. ≥2/1 drinks/d; 0.071. 0.1–1.9/0.9 drinks/d; 0.143. 0 drinks/d.  PA: 0. <75 min/w; 0.25. 75–149 min/w; 0.5. ≥150 min/w.  TV watching: 0. ≥20 h/w; 0.25. 5–19.9 h/w; 0.5. <5 h/w.  BMI: 0. <15 or ≥30 kg/m^2^; 0.167. 15–18.4 or 25–29.9 kg/m^2^. 0.333. 18.5–24.9 kg/m^2^.  Weight gain in last 10 years: 0. ≥10 lb; 0.167. 0–9.9 lb; 0.333. no.  Waist circumference: 0. ≥40.2/34.6 inches; 0.167. 37–40.1/31.5–34.5 inches; 0.333. <37/31.5 inches. | Quantile 1  Quantile 2  Quantile 3  Quantile 4  Quantile 5 | Total energy intake, NSAID/aspirin use, family history of CRC, previous CRC screening via colonoscopy/ sigmoidoscopy, history of polyps, smoking, multivitamin use, supplemental calcium intake, young adult body mass index, menopausal status (women only), and postmenopausal hormone use (women only). |
| Romaguera 2015 | EPIC cohort | 10 European countries | Cohort  (N=3292) | 4.2 | 45.5 | 64.6 (NA) | 872 CRC-specific  mortality | Energy density: 0. >175 kcal/100g/d; 0.5. 126-175 kcal/100g/d; 1. ≤125 kcal/100g/d.  Sugary drinks: 0. >250g/d; 0.5. ≤250 g/d; 1. 0 g/d.  Fruits and vegetables: 0. <200 g/d; 0.25. 200 to <300 g/d; 0.5. ≥400 g/d.  Dietary fibre: 0. ≥25 g/d; 0.25. 12.5 to <25 g/d; 0.5. <12.5 g/d.  Red meat and processed meat: 0. ≥500 g/d or ≥50 g/d; 0.5. <500 g/d and 3 to <50 g/d; 1. <500 g/d and <3 g/d.  Alcohol (Intake the previous day): 0. >30 g/d (men) or >20 g/d (women); 0.5. 20-30 g/d (men) or 10-20 g/d (women); 1. ≤20 g/d (men) or ≤10 g/d (women).  PA: 0. <15 min/d of cycling or sport; 0.5. 15–30 min/d of cycling or sport; 1. manual/heavy manual job, or >2 h/w of vigorous PA, or >30 min/d of cycling/sports.  BMI: 0. <18.5 or ≥30 kg/m^2^; 0.5. 25–29.9 kg/m^2^; 1. 18.5–24.9 kg/m^2^.  Breastfeeding: 0. no; 0.5. 0-6 months; 1. >6 months. | Men:  0–2 points  2.25–2.75 points  3–3.75 points  4–6 points  Women:  0–3 points  3.25–3.75 points  4–4.75 points  5–7 points | Age, year of CRC diagnosis, tumor stage, tumor grade, tumor site, sex, education, and smoking status. |
| Sotos-Prieto 2018 | Nurses’ Health Study； Health Professionals’ Follow-up Study | US | Cohort  (N=87113) | NA | 66.9 | 51.7 (40-75) | 684 CRC-specific  mortality | *Women:*  Score = [1 − 0. 9660 (exp [W− 6.57301)] × 100%, where W= 0.10820 x age + 0.15285 (if past smoker) + 0.90138 (if current smoker) + 0.04676 × BMI – 0.01923 × grams/d of alcohol + 0.0004 × (grams/d of alcohol)2 – 0.029251 × hours/w of physical activity - 0.05113 × diet score  *Men:*  Score = [1 − 0. 96368 (exp [W−7.2437)] × 100%, where W= 0.13580 x age- 0.0005 x (age) 2 + 0.06979 (if past smoker) + 0.42305 (if current smoker) +0.07424 × BMI – 0.00898 × grams/d of alcohol + 0.0001 × (grams/d of alcohol)2 – 0.01755 × hours/w of physical activity - 0.06691 × diet score | Quantile 1  Quantile 2  Quantile 3  Quantile 4  Quantile 5 | Age, race, marital status, baseline postmenopausal hormone use (women only), family history of diabetes mellitus, myocardial infarction and cancer, and baseline history of diabetes mellitus, hypertension, hypercholesterolemia, multivitamin use, aspirin use, energy intake, and physical examination |
| Tabung 2015 |  | US | Case-control  (N=138) | NA | 49.3 | NA (30-80) | 47 CRA | Diet: 0. <5 cups/d FV intake and ≥30% of energy intake from fat; 1. ≥2.5 cups/d FV intake or <30% of energy intake from fat, or both.  Smoking: 0. former or current smokers; 1. never smokers.  Alcohol: 0. >2 drinks/d (men) or >1 drink/d (women); 1. ≤2 drinks/d (men) or ≤1 drink/d (women).  PA: 0. <150 min/w of moderate intensity PA or <60 min/w of vigorous intensity; 1. ≥150 min/w of moderate intensity PA or ≥60 min/w of vigorous intensity PA.  BMI: 0. ≥25 kg/m^2^; 1. 18 to <25 kg/m^2^. | 0-2 points  3-5 points | Age, sex, education, race and reason for colonoscopy, and NSAIDs use. |
| Thomson 2014 | Women’s Health Initiative Observational Study (WHI-OS) | US | Cohort  (N=65838) | 12.6 | 0 | 63.2 (50-79) | 751 CRC  190 CRC-specific  mortality | Diet (diet points): 0. 0-2 points; 1. 3-6 points; 2. 7-9 points.  Alcohol: 0. >1 drink/d; 1. >0– 1 drink/d; 2. nondrinker at baseline.  PA: 0. <8.75 MET-h/w; 1. 8.75–17.5 MET-h/w; 2. >17.5 MET-h/w.  BMI: 0. ≥30 kg/m^2^ at age 18 y, or ≥30 kg/m^2^ at baseline; 2. <25 kg/m^2^ at age 18 y, and <25 kg/m^2^ at baseline; 1. 25 to <30 kg/m^2^ at age 18 y, or 25 to <30 kg/m^2^ at baseline. | 0-2 points  3 points  4 points  5 points  6 points  7-8 points | Age, education, smoking, NSAIDs use at baseline, aspirin use at baseline, unopposed estrogen use, estrogen progestin use, multivitamin use at baseline, race/ethnicity, total energy intake, parous, mammogram, colonoscopy/sigmoidoscopy, family history of cancer, and having a current healthcare provider. |
| Zhang 2018 | Shanghai Men’s Health Study (SMHS) | China | Cohort  (N=59503) | 9.3 | 100 | 55.3 (40-74) | 674 CRC  400 Colon cancer  274 Rectal cancer | Diet (Chinese Food Pagoda score, CHFP): 0. lower two quintiles; 1. upper three quintiles.  Smoking: 0. current smokers or former smoker for <10 years; 1. never smoker former smoker for ≥10 years.  Alcohol: 0. >14 drinks/w; 1. ≤14 drinks/w.  PA (moderate-to-vigorous-intensity exercise participation): 0. <150 min/w; 1. ≥150 min/w.  Waist-hip ratio: 0. ≥0.9; 1. <0.9. | 0-1 points  2 points  3 points  4-5 points | Age, occupation, education, income/person, history of diabetes mellitus, and CRC family history of ﬁrst degree relatives. |

NA: not available; NAA: non-advanced adenoma; AA: advanced adenoma; PA: physical activity; MPA: moderate physical activity; VPA: vigorous physical activity; MVPA: moderate to vigorous physical activity; FV: fruit and vegetable; MET: metabolic equivalent task; MJ: megajoules; NSAID: non-steroidal anti-inﬂammatory drug.

**Supplementary Table 4.** Results of quality assessment

(A) Cohort studies (n=18)

| Study ID | Outcome | Selection | | | |  | Comparability |  | Outcome | | | | | | | Total stars | | Overall quality | |
| --- | --- | --- | --- | --- | --- | --- | --- | --- | --- | --- | --- | --- | --- | --- | --- | --- | --- | --- | --- |
|  |  | Representativeness of the exposed cohort | Selection of the non-exposed cohort | Ascertainment of exposure | Demonstration that outcome of interest was not present at start of study |  | Comparability of cohorts on the basis of the design or analysis controlled for confounders |  | | Ascertainment of outcome | Follow-up long enough for outcomes to occur | | Adequacy of follow-up of cohorts | |  | |  | |  |
| Aleksandrova 2014 | CRC  Colon cancer  Rectal cancer |  | * |  | * |  | * |  | * | | | * | |  | | 5 | | Moderate | |
| Barrubes 2020 | CRC |  | * |  | * |  | ** |  | * | | |  | |  | | 5 | | Moderate | |
| Cheng 2018 | CRC | * | * |  | * |  | ** |  | * | | | * | | * | | 8 | | High | |
| Dartois 2014 | CRC |  | * |  | * |  | ** |  |  | | | * | |  | | 5 | | Moderate | |
| Erdrich 2015 | Colon cancer | * | * |  | * |  | ** |  | ** | | | * | |  | | 8 | | High | |
| Harnack 2002 | Colon cancer | * | * |  | * |  | ** |  | * | | | * | |  | | 7 | | High | |
| Hastert 2016 | CRC |  | * |  | * |  | ** |  | * | | |  | | * | | 6 | | Moderate | |
| Inoue-Choi 2013 | CRC-specific mortality | * | * |  | * |  | ** |  | * | | |  | | * | | 7 | | High | |
| Jones 2018 | CRC  Colon cancer  Rectal cancer |  | * |  | * |  | ** |  | * | | | * | |  | | 6 | | Moderate | |
| Kirkegaard 2010 | CRC  Colon cancer  Rectal cancer | * | * |  | * |  | ** |  | * | | | * | | * | | 8 | | High | |
| Lohse 2016 | CRC-specific mortality | * | * |  | * |  | ** |  | * | | | * | |  | | 7 | | High | |
| Nomura 2016 | CRC  Colon cancer |  | * |  | * |  | ** |  |  | | | * | | * | | 6 | | Moderate | |
| Odegaard 2013 | CRC  Colon cancer  Rectal cancer |  | * |  | * |  | ** |  | * | | | * | | * | | 7 | | High | |
| Petimar 2019 | CRC  Colon cancer  Rectal cancer |  | * |  | * |  | ** |  | ** | | | * | | * | | 8 | | High | |
| Romaguera 2015 | CRC-specific mortality |  | * |  | * |  | ** |  | * | | |  | |  | | 5 | | Moderate | |
| Sotos-Prieto 2018 | CRC-specific mortality |  | * |  | * |  | ** |  | * | | | * | |  | | 6 | | Moderate | |
| Thomson 2014 | CRC  CRC-specific mortality |  | * |  | * |  | ** |  | * | | | * | |  | | 6 | | Moderate | |
| Zhang 2018 | CRC  Colon cancer  Rectal cancer |  | * |  | * |  | ** |  | * | | | * | | * | | 7 | | High | |

(B) Case-control studies (n=8)

| Study ID | Outcome | Selection | | | |  | Comparability |  | Outcome | | | Total stars | Overall quality |
| --- | --- | --- | --- | --- | --- | --- | --- | --- | --- | --- | --- | --- | --- |
|  |  | Is the case definition adequate? | Representativeness of the cases | Selection of controls | Definition of controls |  | Comparability of cases and controls on the basis of the design or analysis |  | Ascertainment of exposure | Same method of ascertainment for cases and controls | Non-response rate |  |  |
| Byrd 2020 | CRA |  |  |  | * |  | ** |  |  | * |  | 4 | Low |
| Carr 2018 | CRC  Colon cancer  Rectal cancer | * | * | * |  |  | ** |  |  | * | * | 7 | High |
| Cho 2019 | CRC  Colon cancer  Rectal cancer |  |  |  | * |  | ** |  |  | * |  | 4 | Low |
| Fliss-Isakov 2020 | CRA |  |  |  |  |  | ** |  |  | * |  | 3 | Low |
| Fu 2012 | CRA | * |  |  | * |  | ** |  |  | * |  | 5 | Moderate |
| Hang 2015 | CRC |  |  | * | * |  | ** |  |  | * |  | 5 | Moderate |
| Hatime 2020 | CRC  Colon cancer  Rectal cancer |  |  |  |  |  | ** |  |  | * |  | 3 | Low |
| Tabung 2015 | CRA | * |  |  | * |  | ** |  | * | ** |  | 7 | High |

(C) Cross-sectional studies (n=2)

| Study ID | Outcome | Selection | | |  |  | Comparability |  | Outcome | | Total stars | Overall quality |
| --- | --- | --- | --- | --- | --- | --- | --- | --- | --- | --- | --- | --- |
|  |  | Representativeness of the sample | Sample size | Non-respondents | Ascertainment of the exposure (risk factor) |  | The subject in different outcome groups are comparable, based on the study design |  | Assessment of the outcome | Statistical test |  |  |
| Erben 2019 | CRA  Advanced colorectal neoplasia |  | * |  | * |  | ** |  | ** | * | 7 | Moderate |
| Knudsen 2016 | Advanced colorectal neoplasia | * | * |  | * |  | ** |  | ** | * | 8 | High |

*CRC: colorectal cancer; CRA: colorectal adenoma
